# Una disertación médica olvidada del siglo XVIII: la “Memoria sobre las virtudes heroicas de la quinua”, de José Manuel Dávalos

**DOI:** 10.1590/S0104-59702024000100062

**Published:** 2024-12-16

**Authors:** Rafael Cerpa Estremadoyro

**Affiliations:** i Profesor asociado, Universidad Nacional Tecnológica de Lima Sur. Lima – Perú rcerpa@untels.edu.pe

**Keywords:** José Manuel Dávalos (1758-1821, Quinua, Medicina botánica, Société Royale de Médecine (Francia, Credibilidad científica, José Manuel Dávalos (1758-1821, Quinoa, Botanical medicine, Société Royale de Médecine (France, Scientific credibility

## Abstract

Se presenta aquí la traducción inédita de un manuscrito en francés, la “Memoria sobre las virtudes heroicas de la quinua”. Mediante ese trabajo, el médico peruano José Manuel Dávalos intentaba ser nombrado socio extranjero en la prestigiosa Société Royale de Médecine, de Francia, a fines de 1787. A pesar de la elogiosa carta de presentación de un diplomático español, la solicitud de Dávalos fue rechazada, posiblemente debido a la falta de credibilidad, en el sentido de David Bloor o Steven Shapin. En la introducción, también se ofrece el contexto histórico en el que se inscribe su ensayo, destacando las características de la institución médica a la que postulaba el doctor limeño.

## Abstract (en)

Presented herein is the unpublished translation of a manuscript in French, the “Memoir on the heroic virtues of quinoa.” Through this work, the Peruvian physician José Manuel Dávalos tried to be appointed as a foreign member of the prestigious Royal Society of Medicine, from France, at the end of 1787. Despite a laudatory letter of introduction from a Spanish diplomat, his application was rejected, possibly due to Dávalos' lack of credibility in the sense of David Bloor or Steven Shapin. The introduction also provides the historical context in which the Peruvian doctor’s work is inscribed, highlighting the central characteristics of the medical institution to which the Limenean doctor sought membership.

En la Biblioteca de la Académie Nationale de Médecine, en París, se conserva un expediente denominado “Fondo Lima”, relacionado con el infructuoso intento del médico peruano José Manuel Dávalos por pertenecer a la Société Royale de Médecine.^1^ Dicho fondo consta de cuatro documentos.^2^ En primer lugar, encontramos una carta de presentación, fechada el 10 de diciembre de 1787 y dirigida al secretario perpetuo de la Société Royale de Médecine, Félix Vicq d’Azyr, un renombrado anatomista y socio de la célebre Académie Royale des Sciences. Quilty Valois, su autor, era un español, probablemente un diplomático, que residía entonces en el Hotel de Embajadores en París.^3^ En la carta, Quilty Valois presenta a los miembros de la asociación parisina el aspirante al título de socio extranjero. La carta iba acompañada de una disertación en francés, sin título, que, por su contenido, podemos denominar *“*Memoria sobre las virtudes heroicas de la quinua” (Dávalos, dic. 1787).^4^ Los otros dos documentos son de uso interno de la Société Royale de Médecine: una pequeña nota donde se señala el objeto de la misiva y otro escrito, el dictamen, de un solo folio, que, aunque no está firmado ni fechado, se presupone es de Antoine-Laurent de Jussieu, otro de los fundadores de la corporación médica y uno de los botánicos más eminentes de su tiempo.

En la carta de presentación, el diplomático destaca la trayectoria y logros del peruano, quien después de abandonar su patria y atravesar una inmensa extensión de mar a riesgo de su propia vida, llegó a Francia. La misiva parece inspirarse, en parte, en el relato que Dávalos hace en el prefacio de su tesis para obtener el bachillerato en medicina: “Movido por el amor a mi patria, y con gran costo de mi fortuna y de mi edad florida, viajé por provincias, reinos, mares y tierras situados bajo otro Sol” (Dávalos, 1787, p.iii). En la Universidad de Medicina de Montpellier, nos dice Quilty Valois (10 dic. 1787), obtuvo el “título de doctor con aplausos debido a sus raras dotes”. El propósito principal de Dávalos, tras su afán de convertirse en socio extranjero de la corporación parisina, era, según el embajador español, ser útil, no solo a su país de origen, sino a la “humanidad entera”, consagrándose a ella. La carta destaca la habilidad del doctor Dávalos como médico y su dedicación a la causa de la medicina, presentándolo como un candidato altamente calificado, y señalando que “la adquisición de un miembro tan adecuado en todos los aspectos resultará, sin duda, de gran utilidad tanto para la botánica como para la historia natural”. No sabemos si Vicq d’Azyr respondió con una carta al embajador, aunque es probable que así fuera. Lo cierto es que, en la parte superior del escrito de Quilty Valois (10 dic. 1787), se lee una escueta indicación: “Decir esto al Dr. Jussieu, responder al Dr. Dávalos”. No es claro quién es el autor de esa directiva, pero debió ser el propio Vicq d’Azyr. En cualquier caso, Jussieu elaboró un breve informe sobre la conveniencia de aceptar el pedido del joven peruano.

Dávalos, en el ensayo presentado a la sociedad parisina, destaca la importancia medicinal de la quinua, una planta común en las “regiones remotas” del Perú. Resalta su eficacia en la cura de diversas enfermedades, basándose en su propia experiencia y en casos específicos que demuestran empíricamente sus beneficios. Aunque ampliamente utilizada como alimento, el limeño pone en relieve sus “virtudes heroicas”, tanto como resolutivo como diurético.^5^ La decocción de esta semilla había demostrado ser efectiva en la cura de contusiones, abscesos internos y pleuresía. Además, es un remedio adecuado contra enfermedades respiratorias y dolores de pecho. Otro aspecto de interés de la disertación es su crítica a los naturalistas que solo buscaban aumentar el número de especies botánicas conocidas, darles un “nombre”, refiriéndose a los grandes proyectos de taxonomía que tuvieron lugar en el siglo XVIII, sin interesarse en sus virtudes terapéuticas. En su ensayo, en tres ocasiones, hace referencia a los “naturales del lugar”, los “indígenas”, que conocían un gran número de plantas heroicas con las que Perú ha enriquecido la medicina. En su tesis, *Sobre algunas enfermedades comunes en Lima y su tratamiento*, Dávalos (1787) menciona otras plantas originarias de América del Sur, como la canchalagua, la ipecacuana o la zarzaparrilla, y sus usos en prácticas curativas. Si bien no hay pruebas de que el conocimiento que manifiesta sobre las propiedades de esas especies vegetales provenga directamente de mediadores indígenas, se tienen indicios de que la información que poseían los llamados “indios herbolarios” ya había permeado a otros sectores de la sociedad peruana en el siglo XVIII.^6^

La elección de la quinua como objeto de estudio en su disertación no debe sorprender. La segunda mitad del siglo XVIII, en la América hispana, se caracterizó por un creciente interés en las ciencias naturales, en particular en la botánica, que se convirtió en un campo fértil para el desarrollo de ideas protonacionalistas. En el caso del Virreinato del Perú, la obra de Hipólito Unanue en el *Mercurio Peruano* sirve de ejemplo para entender cómo la exaltación de las plantas nativas se entrelaza con un sentido emergente de identidad y orgullo nacional. En su artículo “Botánica: introducción a la descripción científica de las plantas del Perú”, de 1791, Unanue, al detallar la riqueza de la flora local, no solo buscaba contribuir al conocimiento científico en general, sino también afirmar la singularidad y el valor del Perú frente a las potencias coloniales europeas. Su enfoque en la botánica como un medio para el avance científico y médico de su patria refleja un compromiso con el suelo patrio, lo que se manifiesta en un protonacionalismo implícito en sus escritos. Este sentimiento de amor patrio también se aprecia en la obra de Dávalos, quien dedicó su tesis de 1787 a la ciudad de Lima y centró parte de su investigación en el empleo de plantas locales para el tratamiento de enfermedades endémicas. El doctor peruano veía su trabajo no solo como una contribución al campo de la medicina, sino también como un “regalo” a su ciudad natal, mostrando implícitamente cómo la ciencia podía servir de vehículo para la creación de una identidad nacional.^7^ En un contexto más amplio, el célebre *Prefacio a la geografía de las plantas de Humboldt*, de Francisco José de Caldas (1849), mártir de la independencia americana, ilustra cómo la botánica se convirtió en una herramienta para el desarrollo de un primer nacionalismo en otras regiones de América. Caldas, al ensalzar la obra de Humboldt, destacaba la importancia de la flora andina y su estudio para la construcción de un sentido de pertenencia y orgullo nacional en Nueva Granada. El naturalista payanés subrayó la relevancia de la quinua como símbolo de la riqueza natural de su suelo natal. La convergencia de estas perspectivas evidencia una tendencia común de emplear la ciencia, y particularmente la botánica, como medio para la construcción de una narrativa nacional y el fomento de sentimientos protonacionalistas.

## La botánica médica en la Société Royale de Médecine

La Société Royale de Médecine, a la que postulaba el joven Dávalos, se había erigido en una de las instituciones “savantes” más importantes del Antiguo Régimen.^8^ Creada oficialmente por cartas patentes en 1778, tenía como principal objetivo mejorar la salud pública, en respuesta a las constantes amenazas de epidemias y epizootias que afectaban regularmente las ciudades y campos de esa parte de Europa. La corporación parisina surgió de la visión compartida por dos destacados hombres de ciencia: Jean-François de Lassonne, primer médico del rey, y Félix Vicq d’Azyr. Bajo la dirección de este último, nombrado secretario perpetuo de la corporación, la sociedad estableció una vasta red de corresponsales en las provincias francesas, quienes servían de informantes, enviando periódicamente reportes sobre enfermedades y epidemias. Además de formar ese equipo de trabajo, Vicq d’Azyr propuso un plan maestro de topografía médica, con el que buscaba analizar la relación entre el hombre y su entorno, consolidando así la posición de la sociedad como una entidad pionera en el estudio y mejora de la salud pública en Francia. El doctor peruano, con su disertación académica sobre las enfermedades endémicas que afligían recurrentemente Lima y que contenía igualmente una introducción que puede considerarse un esbozo de topografía médica, debió haberse sentido particularmente identificado con la misión de esta institución francesa.

Desde su fundación, la corporación francesa se esforzó por proyectarse más allá del ámbito puramente local. La invitación a intercambiar ideas y compartir conocimientos no se limitó solo a los facultativos del reino, sino que se extendió a los “médicos y profesores más célebres de los cursos y universidades extranjeras” (Société..., 1779, p.2). Estos socios extranjeros eran, en su casi totalidad, europeos. Su número estaba fijado en sesenta, según lo estipulado por las cartas patentes, elegidos de entre los profesionales de la salud más renombrados, quienes “se apresuraron a fertilizar los trabajos de la Sociedad” (p.2).

La década de 1770 en Francia se erigió como un período de transición crítica, marcado por el auge de la Ilustración y el creciente cuestionamiento de las estructuras tradicionales del poder. En medio de una crisis económica exacerbada por guerras onerosas y el derroche de la corte, las voces de filósofos como Voltaire y Rousseau resonaban, instando a la libertad y la igualdad y sembrando las semillas del descontento que florecerían luego en la Revolución Francesa. Intentos de reformas agrarias y sociales buscaban aliviar la carga de los campesinos y promover una distribución más equitativa de los impuestos, desafiando así el *statu quo* de la aristocracia y el clero. Este período también fue testigo de avances significativos en la ciencia, con figuras como Lavoisier que transformó la química y adoptó un enfoque cada vez más empírico. En el mundo médico, se observaba una evolución similar con la creación de la Société Royale de Médecine, para contrarrestar precisamente el conservadurismo de las Facultades y “romper el aislamiento de los médicos de provincia” (Meyer, 1972, p.11). La asociación tenía además una impronta ilustrada. En un documento fundacional de la corporación parisina, se subraya la necesidad de la difusión de las “luces” (*lumières*, en un sentido ilustrado) para el progreso de todas las ciencias, pero se destaca que esa exigencia era especialmente apremiante en el campo de la medicina (Société..., 1779, p.vii). A pesar de esto, en 1793, con la llegada de los nuevos tiempos, la otrora institución reformista fue abolida.

Un año después de su creación, la corporación parisina se embarcó en la edición de la *Histoire de la Société Royale de Médecine avec les mémoires de médecine et de physique médicale* (a continuación, *Historia de la Sociedad Real de Medicina*), que llegó a contar con diez volúmenes, publicados entre 1779 y 1798. La institución buscaba así convertirse en un canal para la diseminación del conocimiento médico. El primer volumen estableció el formato y el contenido que iba a tener en lo sucesivo esa publicación. La primera parte, llamada “Historia”, se organizó en doce secciones temáticas. En ella, se colocaron, primero, las notas relativas a la topografía de los diversos cantones franceses y a las enfermedades endémicas y epidémicas presentes en ellos, y, en último lugar, las concernientes a la física médica, siendo la undécima dedicada a la botánica y la historia natural de sustancias vegetales. La segunda parte, llamada “Memorias”, contenía disertaciones escritas tanto por miembros de la sociedad como por médicos y físicos regionales o extranjeros, las que, después de haber sido leídas en las asambleas de la corporación, se juzgaban dignas de ser impresas integralmente. Estas memorias se distribuyeron siguiendo el mismo orden que la primera sección, y abarcaron la misma gama de temas, desde disertaciones meteorológicas y descripciones topográficas, hasta escritos sobre enfermedades endémicas y epidemias, pasando por trabajos de medicina práctica y análisis químico. Es notable la inclusión de artículos sobre epizootias y un buen número de trabajos sobre botánica médica, en los que se estudiaban las sustancias vegetales utilizadas como alimentos o remedios (Société..., 1779, p.ix-x).

Las disertaciones sobre botánica debían cumplir ciertas condiciones para su publicación en la *Historia de la Sociedad Real de Medicina*. Se recomendaba a los eventuales colaboradores que observaran y examinaran especialmente las plantas que crecían en su región, mencionando los nombres que les habían dado tanto los naturalistas como los habitantes del lugar, su etimología cuando era relevante, las propiedades medicinales o económicas atribuidas a estas, así como el tipo de suelo en el que crecían. Estos detalles permitían establecer un registro exacto de las especies vegetales de cada provincia francesa. Por “nombres dados por los naturalistas”, los miembros de la corporación se referían a la nomenclatura polinomial de Tournefort o a la binomial de Linneo (Société..., 1779, p.xix). A partir de esto, se puede concluir que, a diferencia de los grandes taxonomistas de su siglo, los parisinos se interesaban sobre todo en las características de las plantas que representaban una utilidad, tanto para su disciplina como en vista de su explotación comercial. De ahí que se preocupasen también por el tipo de suelo idóneo para su desarrollo, con el objetivo apenas velado de su cultivo en otras regiones. El médico limeño, al redactar su “Memoria sobre las virtudes heroicas de la quinua”, estaba probablemente al tanto de estas directrices. En consonancia con los objetivos de la Société Royale de Médecine, él contrapone un interés puramente taxonómico por las plantas, con un enfoque más bien práctico, centrado en su uso medicinal o alimenticio. En cualquier caso, a excepción del suelo que requiere la quinua, en su ensayo se señalan todas las demás características solicitadas por los parisinos.

## Las razones de un rechazo casi anunciado

A pesar de la elogiosa carta de presentación de Quilty Valois, la candidatura del peruano no fue retenida. No obstante, el dictamen de Antoine-Laurent de Jussieu contiene elementos de gran interés. Dávalos, en el escrito del botánico francés, es un “médico español” que desarrolla su actividad en Lima. Si bien el ensayo ofrece una perspectiva sobre los beneficios potenciales derivados del conocimiento de las plantas peruanas, en especial la quinua, Jussieu (dic. 1787) concluye que es difícil obtener del informe un beneficio sustancial sin que el autor proporcione una elaboración más detallada para hacerlo más útil.

La crítica de Jussieu se centraba en detalles aparentemente “técnicos” del trabajo del doctor limeño. La nomenclatura empleada por Dávalos fue un punto observado. El botánico francés señala que, si bien la quinua es una especie de amaranto, no se trata exactamente del *Amaranthus blitum* (nombre común, “bledo”), como sostiene el peruano en su ensayo. Jussieu, al parecer, no tiene en mente la caracterización de la semilla andina que realizó Louis Feuillée décadas antes en su “Histoire des plantes médicinales qui sont le plus en usage aux Royaumes du Pérou & du Chily dans l’Amérique Méridionale” [Historia de las plantas medicinales más usadas en los Reinos de Perú y Chile en América del Sur] (Feuillée, 1725, p.15) que, de hecho, es más cercana a la clasificación científica actual que sitúa la quinua dentro de las quenopodiáceas.^9^ El error de Dávalos, con todo, era comprensible. Esto se debía en parte a la persistencia de identificar la quinua como una especie de bledo semejante a los de la península Ibérica, primero en los relatos de los cronistas, y luego en las clasificaciones científicas iniciales. Dávalos, que había leído la *Rariorum plantarum historia* [Historia de las plantas raras] de Carolus Clusius (1601, p, p.131), tenía en cuenta que para el sabio flamenco la quinua era un *Blitum Maius Peruvianum*, un bledo, aunque de mayor tamaño que el europeo.

A esto se añade el hecho de que en las primeras ediciones de la *Species Plantarum* de Carl Linneo no figuraba la planta peruana. Fue necesario esperar hasta la célebre cuarta edición, publicada en 1797 por Carl Willdenow, para obtener la clasificación y el nombre científico definitivos de la quinua, a saber, *Chenopodium quinoa W*. Considerando que los socios de la Société Royale de Médecine abogaban por el empleo de la nomenclatura de Tournefort o la de Linneo en las disertaciones sobre botánica, el médico limeño, para cumplir esta recomendación, señaló tentativamente que la quinua era la misma especie vegetal previamente identificada por los naturalistas, incluido Linneo, como *Amaranthus blitum*.^10^ Esto la situaba erróneamente dentro de los bledos. El desacierto del peruano podría atribuirse así a su esfuerzo por cumplir con las “normas editoriales” de la *Historia de la Real Sociedad de Medicina*.^11^

Las otras observaciones críticas apuntaban tanto a la vaguedad al momento de describir las cualidades curativas de la planta como a la forma concisa de presentar hechos que sustentarían esas “virtudes heroicas”. Dávalos presenta las propiedades medicinales de la quinua, aunque de “manera algo imprecisa”. Adicionalmente, Jussieu (dic. 1787) subraya que, para demostrar la eficacia de la decocción de las semillas de quinua, el médico limeño menciona con excesiva brevedad algunos “hechos”, en realidad anécdotas clínicas, de dos pacientes: una mujer de Trujillo aquejada por dolores en la región lumbar y un monje recoleto que sufría de un aparente absceso. Estos casos pueden entenderse como evidencia basada en ejemplos prácticos. Dávalos, no obstante, omite información que sería considerada relevante en la actualidad, como la edad de los pacientes, sus historias médicas o una descripción más detallada de los síntomas.

El botánico francés concluye su dictamen con una frase lapidaria: “El título de asociado extranjero … solo se concede a médicos famosos, conocidos por sus numerosas obras y la reconocida utilidad de estas” (Jussieu, dic. 1787). Ciertamente, entre los socios extranjeros de esa academia, encontramos figuras destacadas de la medicina europea, como William Cullen, quien introdujo una nueva doctrina médica centrada en las disfunciones del sistema nervioso y efectuó la primera demostración pública documentada de la refrigeración artificial o James Lind, el descubridor de la cura del escorbuto mediante el consumo de cítricos. Además, la corporación francesa incluía seis representantes de la península Ibérica, destacando el portugués António Nunes Ribeiro Sanches. Respecto a América, el único representante era el célebre Benjamin Franklin. Aunque más conocido por su trayectoria política y científica, el estadounidense fue incorporado a la Sociedad en 1776, mientras residía en París. Cabe mencionar que poco antes, había sido admitido como miembro de la prestigiosa Académie Royale des Sciences.

Con todo, la razón más importante del rechazo a su candidatura se debería más bien a la ausencia de credibilidad, explicado por Bloor (1991) o Shapin (1994, 1995). No se trataría entonces de un tema que gira exclusivamente en torno a la escasa evidencia empírica aportada por Dávalos, ni a un uso erróneo de la nomenclatura botánica, sino principalmente a factores sociales y culturales. Las ideas, o incluso las personas, adquieren autoridad no solo porque de manera objetiva pertenecen a una comunidad científica, sino también porque se ajustan a los intereses o expectativas de un determinado grupo dentro de esta. La credibilidad de Dávalos pudo haberse visto afectada igualmente por su origen geográfica y culturalmente distante de los centros de poder científico en Europa. La falta de interacción cara a cara, que Shapin (1994) considera como esencial para el reconocimiento de la autoridad y la credibilidad, probablemente jugaron un papel en la percepción de la validez de su trabajo. En una época donde la presentación personal y las introducciones adecuadas eran cruciales para la aceptación en círculos científicos, la distancia y la falta de mediadores confiables pudieron haber sido factores decisivos para Dávalos.^12^ En todo caso, el peruano no era el único hispanoamericano que no satisfacía las altas expectativas de los parisinos. En realidad, la Société Royale de Médecine no contaba con representante alguno de la región. La ciencia, a semejanza de las finanzas, funciona como una economía de crédito y, en el caso de Dávalos, hijo de una nación subalterna, ese crédito era más bien escaso.^13^

Sea como fuere, el ensayo fue considerado insuficiente para otorgar al peruano el preciado título de socio extranjero. Años más tarde, Dávalos ofrecerá una versión diferente de su penoso intento por ingresar a la elite médica europea en su *Alegato en la oposición a la catedra de método de medicina de la Real Universidad de San Marcos de Lima*. La disertación de botánica se transformará en una de química y la negativa se convertirá en una aceptación plena: “Empeño de nuevo la pluma, y una memoria sobre la química me adquiere el distinguido título de socio corresponsal de la Academia Médica de París” (Dávalos, 1810, p.6).





## NOTAS

1 José Manuel Dávalos nació en 1758 en Lima, hijo de Joaquín Dávalos, un criollo de origen quiteño, y María Zamudio, una “morena liberta” de Trujillo (Arias-Schreiber, 1971, p.20). Su vida y carrera reflejan la movilidad y evolución de los “pardos” y mulatos en la última etapa de la dominación española en Perú, mostrando cómo las personas de castas lograron superar ciertas barreras sociales y raciales. Después de una formación inicial en Lima, Dávalos se trasladó a Montpellier para continuar sus estudios médicos. Esta decisión estuvo motivada, en parte, a las restricciones que enfrentaba en el Virreinato del Perú debido a las leyes sobre “limpieza de sangre” que excluían de manera formal a los afroamericanos y sus descendientes de las universidades y profesiones médicas y en parte a su deseo de adquirir una formación médica más avanzada. En la Universidad de Medicina de Montpellier, Dávalos se dedicó al estudio de la medicina bajo la tutela de renombrados maestros como Paul-Joseph Barthez, uno de los fundadores del vitalismo médico, y Antoine Goüan, introductor de la nomenclatura binomial en Francia. A inicios de 1787, defendió una disertación. Su tesis en latín, que abordaba las enfermedades endémicas en Lima, fue bien recibida por la comunidad científica europea. A su regreso a Lima, Dávalos se destacó en la enseñanza y la práctica médica. También mostró un interés particular en la botánica, siendo nombrado profesor interino de esta disciplina en 1796 y más tarde de materia médica en 1809. Dávalos falleció en 1821. Su descanso final fue un modesto nicho en el Cementerio General de Lima. Varios historiadores han estudiado la vida de nuestro autor, entre ellos Polo (1885); Lavalle (1887); Vargas Ugarte (1943); Lastres (1951), quien se apoyó considerablemente en los dos primeros. En dos libros de reciente publicación, se ofrecen datos biográficos de Dávalos. Aunque se centra en los debates sobre la reforma médica y el crecimiento demográfico en el Virreinato del Perú durante el periodo borbónico, Warren (2010) documenta también las actividades de médicos y cirujanos negros, incluido Dávalos. Por su parte, Jouve Martín (2014) analiza las contribuciones de tres médicos afroperuanos, incluyendo a Dávalos, en el ámbito de la medicina y salud pública de la Lima colonial y de inicios de la república, mostrando cómo superaron obstáculos raciales y sociales. Si bien no se centra exclusivamente en nuestro autor, Jouve Martín aporta perspectivas sobre él, pese a repetir algunos errores cometidos por biógrafos anteriores. Respecto al tema de la “limpieza de sangre” y la exclusión de zambos, mulatos y cuarterones de la Universidad de Lima, consúltese Lanning (1967, 1985) y Jouve Martín (2014).

2 En la Biblioteca de la Academia de Medicina, el Fondo Lima se registra como SRM 135A dossier 9. Es importante señalar que la disertación de Dávalos, en el manuscrito original en francés, no cuenta con un título. Sin embargo, considerando el contenido del texto, podría titularse “Memoria sobre las virtudes heroicas de la quinua”. Cabe destacar que, en dicho fondo, la disertación sin título de Dávalos es llamada de manera algo equívoca como “Memoria sobre las plantas del Perú”.

3 Al estar firmada solo con su apellido, no es claro cuál de los Quilty Valois, una familia de ricos comerciantes de origen irlandés, establecidos en Málaga, fue el autor de la misiva. Probablemente, se trate de Mateo Esteban Quilty y Valois, nacido en Málaga en 1738 y fallecido en París en 1799. En 1791, don Mateo se desempeñó como diplomático español en Francia durante la Revolución Francesa (Fernández de Angulo, Cólogan, 2022, p.101).

4 Dávalos utiliza el epíteto “heroico” en varios pasajes de su ensayo, para resaltar la intensidad y la eficacia sobresaliente de ciertos remedios. Según el *Nouveau dictionnaire universel de la langue française*, un medicamento era calificado como “heroico”, cuando se consideraba que poseía propiedades excepcionales, capaces de combatir enfermedades con fuerza y eficacia notables (Poitevin, 1868, p.21). Este calificativo no solo subraya la potencia excepcional de la sustancia, sino que incluso refleja un cierto grado de admiración hacia su capacidad curativa.

5 En el volumen sexto de su *Diccionario de medicina y cirugía o biblioteca manual médico-quirúrgica*, Antonio Ballano (1807, p.382) aborda el concepto de los resolutivos dentro de la materia médica, definiéndolos como aquellos medicamentos capaces de “disipar los humores que estorban a las partes, y las estiran contra el orden natural”. Esta definición refleja la teoría humoral que aún tenía influencia en la medicina en aquel entonces, donde la salud se entendía como un equilibrio entre los diferentes humores del cuerpo. La resolución de tumores, según Ballano (1807, p.382), representa la terminación más favorable de los tumores preternaturales, destacando que, en ciertos casos, como los tumores críticos, era preferible la supuración para prevenir que el humor morbífico reingrese en la sangre y cause daños mayores en partes internas.

6 Véase lo afirmado por Hipólito Unanue (29 mayo 1791), otro renombrado médico peruano, sobre los saberes indígenas en materia médica en su artículo “Botánica: introducción a la descripción científica de las plantas del Perú”, publicado en el *Mercurio Peruano*.

7 En múltiples ocasiones, Dávalos emplea el término latino “patria” en su tesis. En la introducción de esta, enfatiza su deseo de proponer un tema que fuera útil y beneficioso para los peruanos, mostrando una vez más su compromiso con su patria: “Nada se me presentó que respondiera mejor a mis deseos que intentar [hacer] un pequeño estudio sobre las enfermedades familiares de mi patria” (Dávalos, 1787, p.5-6).

8 Sobre la Real Sociedad de Medicina, además de consultar las propias obras de la corporación francesa, resultan de gran utilidad las referencias de Peter (1967) y Gramain (2013).

9 La “Historia de las plantas medicinales” de Louis Feuillée está contenida en el tercer volumen de su *Journal des observations physiques, mathematiques et botaniques, faites par ordre du roi sur les côtes orientales de l’Amerique Méridionale, et aux Indes Occidentales* [Diario de las observaciones físicas, matemáticas y botánicas realizadas por orden del rey en las costas orientales de América del Sur y en las Indias Occidentales]. Según el naturalista francés, la quinua es una planta anual que alcanza una altura de aproximadamente dos pies y presenta hojas similares a las del *Chenopodium pes anserinus* (Feuillée, 1725, p.15-16).

10
Linneo (1753, p.990) describe esta especie de planta en las primeras ediciones de su *Species Plantarum.*

11 El empleo correcto de la nomenclatura de Linneo constituía un elemento fundamental para la legitimación del conocimiento científico en ese tiempo. El caso del cirujano Francisco Antônio de Sampaio, detallado por Nogueira y Kury (2022), ilustra de manera elocuente los desafíos que enfrentaban aquellos cuyos trabajos se desarrollaban al margen de este paradigma. A pesar de sus exhaustivos estudios sobre las plantas de la capitanía de Bahía y sus aplicaciones médicas, Sampaio no logró el reconocimiento de la comunidad científica de Lisboa, precisamente por carecer de la formación y las condiciones necesarias para trabajar siguiendo los lineamientos de la botánica de Linneo.

12 No es claro si el factor racial tuvo alguna implicancia en el rechazo de la candidatura de Dávalos como miembro asociado de la institución parisina. En su carta, Quilty Valois lo presenta, a los miembros de la academia, de manera bastante neutra, señalando simplemente que el señor Dávalos era un doctor en medicina originario o asentado en la ciudad de Lima. Adicionalmente, en la nota escrita quizás por Vicq d’Azyr, se señala que Dávalos era un “médico español [que se encontraba] en Lima”.

13 Si bien Benjamin Franklin era el único representante americano en la Societé Royale de Médecine, su caso no es comparable al de Dávalos. En el momento que fue incorporado a la corporación parisina, se desempeñaba como embajador de los EEUU en Francia y ya contaba con un enorme prestigio antes de su incorporación. En el caso de los socios extranjeros de la Societé Royale de Médecine, originarios de España, los médicos Juan Gámez, Jaime Bonells, Casimiro Gómez Ortega, Mucio (o Muzio) Zona y José Amar y Arguedas (Société..., 1779, p.32), todos ellos pertenecían a la élite médica española. A los méritos científicos individuales, se añaden la pertenencia a instituciones académicas prestigiosas, el servicio a la monarquía en tanto médicos de cámara, y un papel activo en el sistema del protomedicato, factores todos que pueden considerarse como criterios de selección para pertenecer a la élite médica de la época. Por ejemplo, la carrera de Casimiro Gómez Ortega, marcada por su amplio conocimiento y contribuciones en botánica, farmacia y medicina, así como su rol en la creación y participación en la Real Academia Médica Matritense, demuestra cómo el mérito científico y académico, complementado con la pertenencia a redes académicas clave, constituía una base importante para el reconocimiento internacional. La relación con la corte real y el protomedicato también emerge como un criterio significativo para el ascenso a la élite médica, como lo ilustra la carrera de José Amar y Arguedas, médico de los reyes Fernando VI y Carlos III, y protomédico de Navarra.
