# Supplementary material for: A forgotten medical dissertation from the eighteenth century: the “Memoir on the heroic virtues of quinoa,” by José Manuel Dávalos
Source: Hist Cienc Saude Manguinhos. 2024 Dec 16;31:e2024062. [Article in Spanish] doi: 10.1590/S0104-59702024000100062 (PMC11654732; doi:10.1590/S0104-59702024000100062)
Supplement: Supplementary file 1 [file 0104-5970-hcsm-31-e2024062-suppl01.pdf]

[Memoria sobre las virtudes heroicas de la quinua]

[f.º 1]

Entre el inmenso número de especies de plantas que la naturaleza parece haber multiplicado prodigiosamente, muchas sirven de alimento, otras como medicinas, y otras son altamente venenosas. Es importante ocuparse seriamente de conocer unas y otras, y poner al alcance de todos la capacidad de distinguirlas. El estudio de estas plantas, tan interesante como útil, dio a la medicina un nuevo brillo y prestará a la humanidad un verdadero servicio. Desde los comienzos de la botánica, debemos a los trabajos de naturalistas infatigables el conocimiento de un número casi ilimitado de plantas, número que aumenta cada día. Estos nos permitieron conocer algunas útiles, venenosas etc., pero como su principal objetivo era únicamente ponerles un nombre, solo por casualidad, o por el relato de los naturales[1] de los países que visitaron, llegaron a conocer sus virtudes. Hay que admitir, sin embargo, que ha habido botánicos celosos por el incremento de la ciencia que realizaron pruebas con animales y consigo mismos para descubrir las virtudes de algunas plantas. ¿Debemos confiar en las experiencias realizadas en animales? Porque la analogía no siempre es perfecta, y hay plantas que, siendo venenosas para unos, son un alimento saludable para otros. Además, hay algunas que son contravenenos para ciertos animales, y que únicamente el instinto los lleva a buscarlas, cuando necesitan usarlas. Es deseable que esta especie de impulso que anima hoy a los naturalistas de los países cultos de Europa y que les hace recorrer todas las partes del mundo para el avance de la historia natural, es deseable, digo, que este valioso impulso los motive a informarse sobre las virtudes de las plantas con los naturales de los países visitados. Parece que la naturaleza en cada suelo solo revela sus [secretos] a aquellos que lo habitan. Nada demuestra mejor lo que decimos que el gran [f.º 2] número de plantas heroicas con las que Perú ha enriquecido la medicina, cuyo conocimiento se debe solo a los originarios de esta región, como la quinua, la ipecacuana, la zarzaparrilla, la calaguala (planta de la que hablaré en un escrito), la canchalagua, el culén, entre otras. Pero lo que me confirma asombrado en mi opinión es el inmenso número de plantas heroicas que utilizan hoy estos desdichados peruanos, sin que ni los habitantes educados del Perú ni los viajeros extranjeros enviados por los gobiernos español y francés hayan hecho el menor esfuerzo por conocerlas. Sus miras y cuidados se limitan a recorrer el campo y recolectar las plantas que se encuentran allí, mientras que los desafortunados peruanos, agobiados por toda clase de males, bajo una tiránica opresión tan injusta como opuesta a las sabias intenciones del soberano, gimiendo sin ayuda humana alguna, curan sus males con plantas. Los sabios naturalistas, deslumbrados por la vana gloria de aumentar

el número de plantas conocidas, posteriormente les atribuyeron virtudes que no existen y trataron de suplir la realidad con supuestos que la naturaleza niega. Esto demuestra, de manera concluyente, cuánto descuidaron estos sabios naturalistas el conocimiento útil de las virtudes de las plantas, como lo prueba el silencio guardado hasta ahora por los viajeros científicos que han recorrido esa región sobre la virtud de la planta que es objeto de esta disertación. En efecto, nada más común en esas regiones remotas que la quinua, planta conocida por los naturalistas con el nombre de *Amaranthus blitum*. Carolus Clusius la menciona, aunque solo para decir que los habitantes de Quito se sirven de ella como alimento. De hecho, se la usa mucho como alimento, siendo muy saludable, pero es en la medicina que su empleo es [más] importante. Como mi propósito principal en este trabajo es hablar de esta planta como medicamento, será suficiente con nombrarla, ya que el tiempo no me permite hacer una descripción de ella, tarea que reservo para una ocasión más propicia. La planta de la que hablo es, pues, el *Amaranthus blitum linnea*, conocida por los naturales como *quinua dulce*, porque hay otra llamada *amarga*, una de las veintitrés especies sin duda conocidas por Linneo. Sus virtudes más destacadas son la de ser un resolutivo heroico, un grave [¿incisivo?][2] y un diurético admirable. Es importante destacar que no se utiliza la planta en sí misma, sino la semilla. [f.º 3]

Estos médicos la emplean con gran éxito en todos los casos [de] contusiones, abscesos internos y pleuresía. En el primer caso, tiene un efecto casi inmediato y sin ninguna recaída, lo que ha llevado a los médicos a denominar a la decocción que [se extrae] de esta semilla como bebida *contra casum*.[3] En los abscesos internos, actúa con igual eficacia, desapareciendo el absceso casi de manera imperceptible tras quince días de tomar la decocción por la noche y por la mañana. Durante ese tiempo, la orina es más abundante, y si no lo es, el sudor sí lo es. En fin, también libera rápidamente a los niños de esta misma enfermedad, que apenas deja tiempo para consultar al cirujano, quien entonces no dispone de otra solución que el triste recurso del hierro.

Si en dichas enfermedades la decocción de quinua es un remedio altamente eficaz para combatirlas, también lo es en la pleuresía y la peripneumonía. Es tan eficaz que el pueblo, tan pronto como siente algún malestar en el pecho, como resfriados (que no son raros) o dolores, recurre inmediatamente a esta decocción, que se prefiere sobre una hierba llamada "del Paraguay" (véase mi *Specimen de morbis Limanis*, capítulo noveno, sobre la enfermedad de siete días, p.133), ya que de esta forma la transpiración se intensifica y con ella logran combatir efectivamente el preludio de una enfermedad más grave.

Todo lo que he relatado sobre las asombrosas virtudes de esta planta se basa no solo en los relatos y la fe de otros, sino que yo mismo he tenido la feliz experiencia durante los cinco años que ejercí la medicina como primer médico de hospitales. Nunca me ha fallado y el éxito siempre ha estado en línea con las expectativas y las indicaciones bajo las cuales la he administrado. Lo que demuestra de forma incontrovertible la veracidad de mis afirmaciones son los siguientes dos casos. Un monje recoleto fue atacado en la región hipogástrica por dolores y [mostraba] todos los síntomas, lo que llevó al médico a sospechar la existencia de un absceso interno. Se consultó a los cirujanos, que sugirieron, como de costumbre, realizar una punción para verificar la presencia de pus. Mi opinión fue completamente opuesta. Aconsejé no realizar intervención alguna y evitar el uso del hierro, optando en

cambio por tratar la enfermedad con la mencionada decocción de quinua y todos los demás medios auxiliares apropiados para tales casos. Siguiendo mi recomendación, después de tres meses, el paciente se recuperó completamente.

Una señora de treinta años que vivía a cien leguas de Lima, en la ciudad de Trujillo, sufría de fuertes [f.º 4] dolores en la región lumbar. La escasez de médicos en aquella región y los consejos imprudentes de gentes ignorantes y supersticiosas llevaron a creer que estaba embrujada. En consecuencia, le administraron medicamentos acordes con esa idea. Tras experimentar la total ineficacia [de este enfoque], ella envió a la Facultad de Lima una consulta sobre su enfermedad elaborada por un médico recién llegado a esa región. Después de una discusión detallada, los médicos consultados acordaron unánimemente que la causa de la enfermedad debía ser el residuo de una inflamación mal curada. En consecuencia, recomendaron tratar la enfermedad con la decocción de semillas de quinua edulcorada con jarabe de quinina, dejando a criterio del médico tratante la aplicación de otros tratamientos adecuados para un caso semejante. Este consejo se siguió al pie de la letra, y después de seis meses recibimos la feliz noticia [de] la recuperación casi completa del paciente. No se puede insistir lo suficiente sobre la virtud heroica de la semilla de quinua; al presenciar los prodigiosos efectos de esta planta, uno se convence de su enorme valor. Sería deseable introducir la planta en esta región, ya que está ausente entre todas las especies de *Amaranthus* que se cultivan en el Jardín del Rey. También sería provechoso verificar el efecto descrito y del cual he sido testigo.

Hace dos años, conocí en Montpellier a un portugués que había viajado allí para recibir tratamiento y que había pasado algún tiempo en Perú, incluso en Lima. Habiendo ido la casa del renombrado cirujano de la región, Vigar[o]us,[4] donde él se hospedaba, y encontrándome allí para visitarlo, después de nuestra conversación habitual, él me dijo: "Aquí está", mientras me mostraba una bolsa, "aquí está el verdadero bálsamo de la vida, [que] me la ha devuelto tres veces". Movido por la curiosidad, examiné el contenido de la bolsa. No encontré otra cosa que la semilla de *Amaranthus blitum* o quinua dulce, de la cual tomé un puñado para llevárselo al famoso botánico Goüan.[5]

Les ruego, señores, que sean indulgentes con un extranjero que se compromete a redoblar sus esfuerzos para contribuir útilmente a la sociedad. Me despido con el mayor respeto.

Señores,
P.D. Solicito el título de asociado extranjero.

Su muy humilde y obediente servidor, Dávalos D., de las universidades de Lima y Montpellier y miembro asociado de la Academia de Lima.

[Firma]

n.14

NOTAS

[1] [Nota de Rafael Cerpa Estremadoyro] En el original francés, aparece el término "indigènes". Dávalos emplea esta palabra en otras dos ocasiones en este ensayo. En cada caso, hemos optado por traducir esa palabra por "naturales".

[2] [Nota de Rafael Cerpa Estremadoyro] Texto de difícil lectura en el original francés. Al parecer, Dávalos emplea el epíteto "incisivo" o "incindente". En la materia médica del siglo XVIII, los medicamentos incindentes tenían la capacidad de atenuar, dividir y fundir humores espesos, cuajados y coagulados. Antonio Ballano (1807, p.189), en su *Diccionario de medicina y cirugía o biblioteca manual médico-quirúrgica*, describe estas sustancias, considerándolas más enérgicas y activas que los simples aperitivos (medicamentos que no producen evacuación alguna). Según Ballano, los incindentes no solo tienen una acción más fuerte y penetrante en la disolución de humores en obstrucciones, sino que también provocan en los sólidos "oscilaciones más vivas y más repetidas", diferenciándose así de otros medicamentos por su sabor más fuerte y su eficacia en eliminar obstrucciones e infartos en las vísceras del vientre (p.189).

[3] [Nota de Rafael Cerpa Estremadoyro] Expresión latina que puede traducirse como "contra la caídas".

[4] [Nota de Rafael Cerpa Estremadoyro] Se trataría de François Vigarous, entonces profesor de la Universidad de Medicina de Montpellier. Formó parte del jurado en el examen del peruano para la obtención del bachillerato. Véase Dávalos (1787, p.ii).

[5] [Nota de Rafael Cerpa Estremadoyro] Antoine Goüan (1733-1821) fue catedrático de botánica en la Universidad de Medicina de Montpellier. Es reconocido por ser el primero en emplear en Francia la nomenclatura binomial de Linneo. También fue parte del jurado en el examen de Dávalos.
